# Minimally Invasive Embedding of Saturated MSU Induces Persistent Gouty Arthritis in Modified Rat Model

**DOI:** 10.1155/2021/6641701

**Published:** 2021-06-08

**Authors:** Han-Lin Xu, Sheng-Kun Li, Xiao-Ao Xue, Zi-Yi Chen, Ying-Hui Hua

**Affiliations:** Department of Sports Medicine, Huashan Hospital, Fudan University, No. 12 Urumqi Middle Rd., Shanghai 200040, China

## Abstract

**Introduction:**

Animal models are valid for *in vivo* research on the pathophysiological process and drug screening of gout arthritis. Intra-articular injection of monosodium urate (MSU) is the most common method, while stable MSU deposition enveloped by inflammatory cells was rarely reported.

**Objective:**

To develop a modified gouty arthritis rat model characterized by intra-articular MSU deposition and continuous joint pain with a minimally invasive method.

**Method:**

A total of twenty-four rats were randomly allocated into six groups. Three intervention groups of rats received intra-articular MSU embedment. Sham groups received pseudosurgeries with equal normal saline (NS). Gross parameters and pathological features of synovium harvested from anterior capsule were estimated. Mechanical pain threshold tests were conducted over a 96-hour period postoperatively. Moreover, quantitative immunofluorescence was conducted to assess tissue inflammation.

**Result:**

After MSU embedding, rats got more persistent arthritic symptoms as well as tissue MSU deposition. More significant synovial swelling was detected in the MSU group compared to sham groups (*P* < 0.025). Behavioral tests showed that the embedding of MSU resulted in prolonged mechanical hyperalgesia during 2 hours to 96 hours postoperatively (*P* < 0.05). MSU depositions enveloped by inflammatory cells that express IL-1*β* and TNF-*α* were detected in embedding groups. Quantitative immunofluorescence suggested that the frequencies of MSU interventions upregulated expression of proinflammatory factors including IL-1*β* and TNF-*α* (*P* < 0.05).

**Conclusion:**

A minimally invasive method was developed to establish modified rat model of intra-articular MSU deposition. This model was proved to be a simple reproducible method to mimic the pathological characteristics of persistent gouty arthritis.

## 1. Introduction

Gout is a systematic disease of uric acid disturbance with increasing incidence and burden currently [1–3]. The central pathological mechanism of gout refers to increased serum uric acid concentration (more than 6.8 mg/dl) and crystals deposition in joint cavities [3]. Oversaturated urate clusters, nucleates, and grows in synovial fluid and deposits on surface of the cartilage, synovium, or tendons [4, 5].MSU crystal in synovial fluid induces neutrophil-dominated inflammation characterized by typical NET structure of MSU crystals enveloped densely by neutrophils [6, 7]. Structures in joints were gradually eroded by repeated inflammatory response, resulting in joint pain and impaired function [8].

Previous animal models of gout are mainly classified into a hyperuricemia model, local injection model, and gene knockout model [9, 10]. Intra-articular or subcutaneous MSU suspension was injected to mimic gout flares, which was proved feasible for researches on local inflammatory response [11–14]. However, it was proved that uricase, which metabolizes and excretes urates rapidly, is present in most common mammal experimental animals including rats, mice, or rabbits. Hence, since self-limiting symptoms and pathological findings are dominated by acute inflammatory cell infiltration, the injection model was used primarily to measure the therapeutic effects of acute inflammation and anti-inflammatory drugs [11, 15, 16] Wu et al. introduced a method of homologous recombination in mouse embryonic stem cells to establish a mouse model lacking urate oxidase [17]. Congenital MSU deposition in the kidney was observed in mutated mice, but the mortality rate exceeded 50 percent in the first four weeks after birth. Due to the low survival rate, the difficulty of gene editing, and cost of experiment, it is hard to be comprehensively applied for further studies. Other researchers performed intraperitoneal or subcutaneous injection of MSU to simulate gout synovitis and have achieved good results, but this air-pouched method is not suitable for the study of chronic gouty arthritis focusing the changes of the synovial membrane, cartilage, and bone [18–20].

In this report, we assume multiple interventions with sufficient MSU could simulate the pathophysiological process of recurrent gout flares even when uricase is not inhibited. By redesigning approach and frequency of MSU embedment, we demonstrated that MSU embedment resulted in persistent symptoms, including tissue swelling and mechanical hyperalgesia in rat. Moreover, pathological changes manifested as crystal deposition densely enveloped by inflammatory cells that express IL- (interleukin-) 1*β* and TNF- (Tumor Necrosis Factor-) *α* were detected as well. This report indicates a producible, economical, and accurate model for in vivo studies on the deposition of MSU and local structure erosion in the future.

## 2. Methods

### 2.1. Animal Study

**Figure 1:**
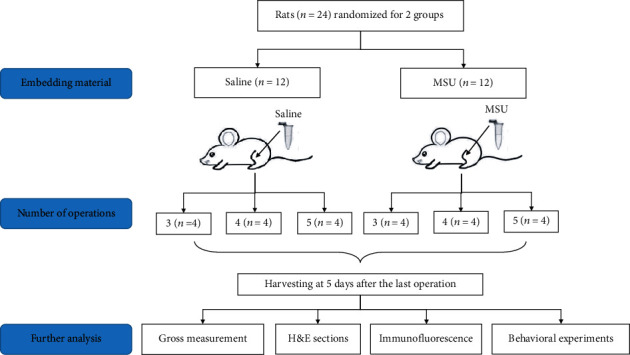
Flow diagram of animal study.

A total of twenty-four male SD rats (Shanghai Lab. Animal Research Center, Shanghai, China) aged 8 weeks and weighted from 160 to 180 grams were used for the present study grams. Experimental procedures passed a review by the Animal Welfare and Ethics Group, Department of Experimental Animal Science, Shanghai Medical College of Fudan University, Shanghai, China (Approval Number 2019020405). The MSU crystal was prepared by pH titration of 99% uric acid (Sigma-Aldrich, USA) according to the method proposed by a previous study [12]. A MSU crystal suspension of 100 mg/ml was prepared then. All the rats were randomly separated into four groups. MSU suspension was embedded into joint cavity at 0.2 ml in MSU intervention groups, respectively, while 0.2 ml normal saline (NS) was injected into the joint cavity in sham groups. Minimally invasive procedures were performed three to five times for each rat, depending on the group, and the intervals between operations were five days. (Figure 1).

**Figure 2:**
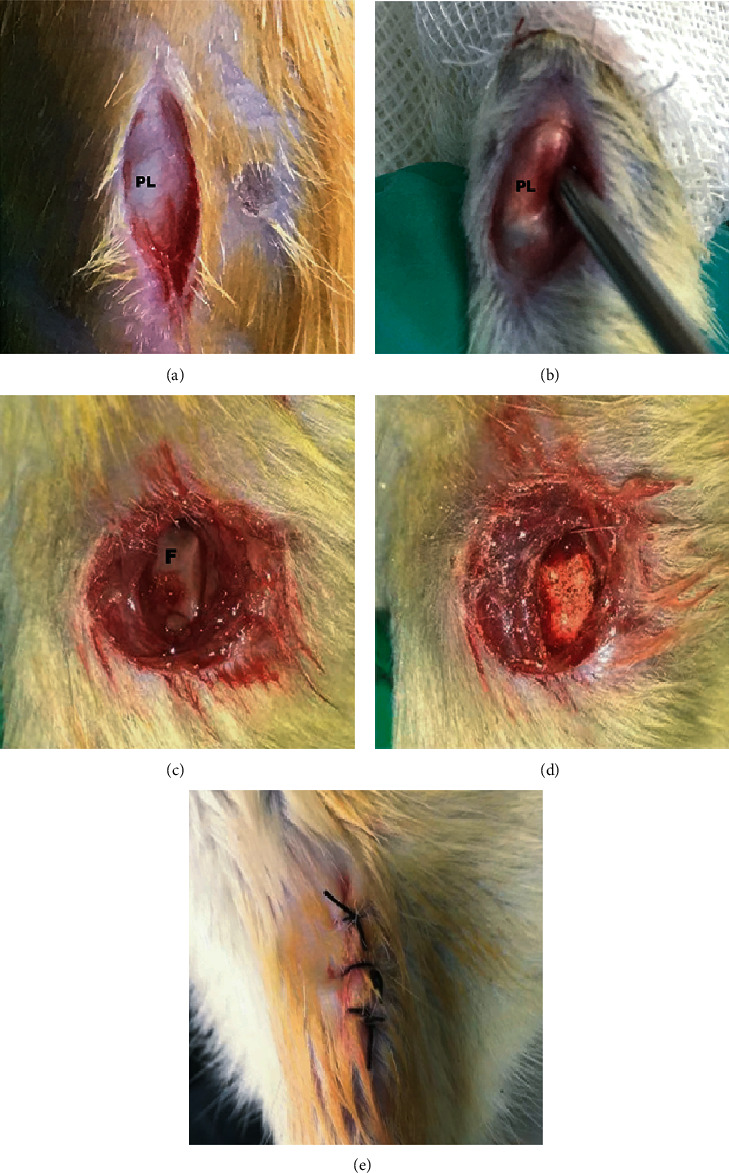
Operating procedures of MSU embedment on rats. Protocol for establishing MSU embedment models. (a, b) The skin and capsule of the joint were incised layer by layer along the lateral side of the patellar ligament, and the joint cavity of the knee was exposed. (c) The intercondylar fossa cartilage of femur was damaged by a Kirschner wire. (d) The MSU crystal was embedded. (e) The incisions of the articular capsule and skin were closed. PL: patellar ligament; F: femur.

The animals were housed in plastic cages in a 12-hour light and dark cycle with free access to food and water. Anesthesia was induced by inhaling 3% isoflurane and maintained by inhaling 1.5% isoflurane. Bilateral knee joints of each rat were operated under anesthesia. An 8 mm incision was made on the lateral side of the joints. The joint capsule was incised longitudinally lateral to patellar tendon to expose the femoral trochlear. A chondral defect was drilled by a Kirschner wire (1.5 mm in diameter, 2 mm in depth) in the femoral trochlear groove. All incisions were cleaned and sutured after surgeries, and there were no restrictions on animal activities (Figure 2).

The dose and the test period are determined based on the preliminary experiments. Longer intervention interval could lead to poor deposition effect, while shorter intervention intervals are detrimental to animals' recovery and welfare. The 5-day interval ensures the healing of the incision. The experimental results also showed that the activity and 50%PWT of the sham group recovered normal 5 days after surgeries.

### 2.2. Gross Measurement

Rats were sacrificed by cervical dislocation on the five days after the last surgery. The synovium of joint cavity was dissected and harvested for further analysis. The width of the patellar ligament and the thickness of the synovium were measured by a micrometer caliper (I. QUIP, China) to evaluate effects of MSU on local tissues. The anterolateral synovium (5 mm × 5 mm) on the lateral side of the patellar tendon is harvested, and the area of incisions and sutures is avoided. The area below the patella is used as the patellar tendon sampling area (10 mm × 3 mm). Each specimen will be measured three times and averaged as the final result. All gross measurements are performed and recorded by a blinded researcher.

### 2.3. Histologic Examination

The tissues were fixed in 4% paraformaldehyde. Dehydration was processed and embedded in paraffin. The synovium rats were processed for H&E staining and immunofluorescence staining as previously described [21]. For immunofluorescent staining, the synovium specimens were stained for IL-1*β* (ProteinTech, 16806-1-AP) and TNF-*α* (ProteinTech, 60291-1-lg). The required reagents were purchased from Sigma-Aldrich, St. Louis, MO, USA, and Servicebio, Wuhan, China. An ortho-fluorescent microscope (Nikon, Japan) and image system (Nikon, Japan) were used for imaging of samples.

For quantitative analysis, Image-Pro Plus 6.0 software (Media Cybernetics, Inc., Rockville, MD, USA) was used to convert green/red fluorescence monochrome photos into black and white images, and then the unified standard for judging all positive photos was determined. The integral optical density (IOD) and pixel AREA of tissues of positive images were obtained by analyzing each photo. The average optical (AO) density value was calculated, and AO = IOD/AREA. The higher the AO value indicated, the higher the positive expression level.

### 2.4. Behavioral Tests

The mechanical allodynia was determined using a series of calibrated von Frey filaments (North Coast, USA). Fifteen minutes before the test, the rats were individually habituated in a transparent plexiglass chamber on an elevated mesh floor. The filaments were applied perpendicularly to the plantar surface of the hind paw until the filaments buckled slightly for 3 seconds. A sharp withdrawal of the paw and licking in response to the application or removal of stimulation were considered positive response [15, 16]. The paw withdrawal threshold (PWT) was determined using the Up-and-Down Method as described in previous studies [22].

After measuring the baseline (6.33 ± 1.58 g) in the preliminary experiment, the fiber filaments of 4 grams were selected as the first stimulus. In formal trials, rats that did not respond to filaments of 16 grams or more were excluded. This is because when the filaments stimulated more than 10% of the rats' body weight, the stimulation was more likely lifting the hindfoot than stinging it. All behavior tests are performed and recorded by an independent researcher, and the interventions on rats were blind to her.

### 2.5. Statistical Analysis

Statistical analyses were performed by GraphPad Prism 8.0.1 software. All datasets were tested for normality for a *t*-test, and if the normality test failed, the Mann–Whitney rank-sum test was used for an intragroup comparison. Results are expressed as the mean ± SD. *P* value < 0.05 is considered significant.

## 3. Result

All the rats received surgical interventions according to the experimental protocols. No incision-related infection, MSU leakage, or other complications occurred.

**Figure 3:**
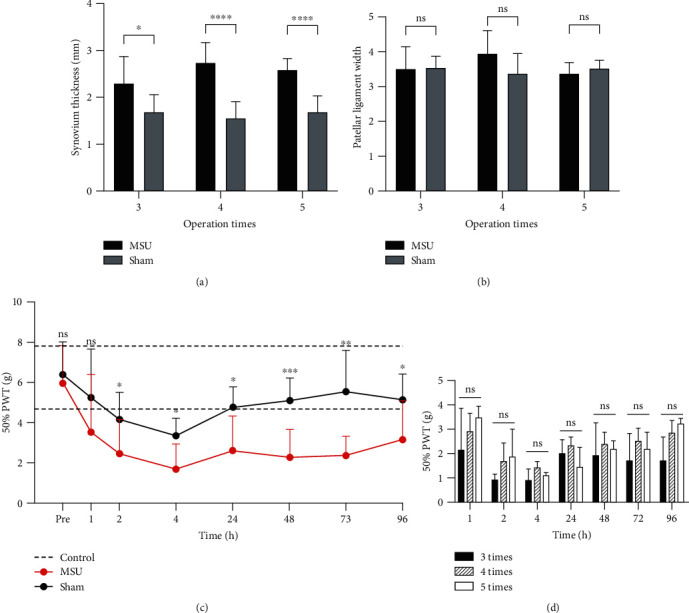
Gross changes in joint tissue and mechanical pain threshold in rats after MSU embedding. (a) The thickness of the synovium lateral to the patellar ligament in the rats. The synovium thickness of the MSU groups was greater than that of the sham groups (*n* = 8 for each group; two-tailed *t*-test, ^∗^*P* = 0.0250, ^∗∗∗∗^*P* < 0.0001). (b) No significant (NS) differences were detected between the sham groups and MSU groups (*n* = 8 for each group; two-tailed *t*-test). (c) The 50%PWT of the wild-type littermates (*n* = 8) is shown between the dotted lines (6.33 ± 1.58 g). The pain threshold of the MSU groups was significantly lower than that in the sham group within 96 hours (*n* = 7 for sham group; *n* = 10 for MSU group; the two-tailed Mann–Whitney test was applied in the time point of 4 hours and 24 hours; a two-tailed *t*-test was applied for other time points; Pre: *P* > 0.05; 1 hour: *P* > 0.05, 2 hours: *P* = 0.0461, 4 hours: *P* = 0.0164, 24 hours: *P* = 0.0311; 48 hours: *P* = 0.0005; 72 hours: *P* = 0.0010; 96 hours: *P* = 0.0355). (d) No significant differences were detected between the MSU groups with different operation frequencies at all time points (*n* = 4 for each group; one-way ANOVA was applied for each time point; *P* > 0.05 at all time points with different operation frequencies). The *x*-axis represents time points before and after operations. Data shown as the mean ± SD.

Gross measurements and behavioral tests were conducted to evaluate the local effects of MSU-mediated inflammation. Significant synovial swelling was detected in the MSU group compared to the sham group five days after the last intervention (*P* = 0.0250 after three interventions, *P* < 0.0001 after four or five interventions) (Figure 3), while patellar ligament was not affected by MSU inflammation (*P* > 0.05 in all groups).

Behavior tests revealed abnormal reductions of the PWT in MSU groups compared to the sham groups started with 2 hours after the intervention (*P* < 0.05). Within 24 to 48 hours after the operation, the pain threshold of the rats in the sham groups gradually returned to the normal level. The pain threshold of the MSU groups also recovered, but it was significantly lower than that in the sham group within 96 hours (*P* < 0.05 during 2 hours to 96 hours postoperatively).

**Figure 4:**
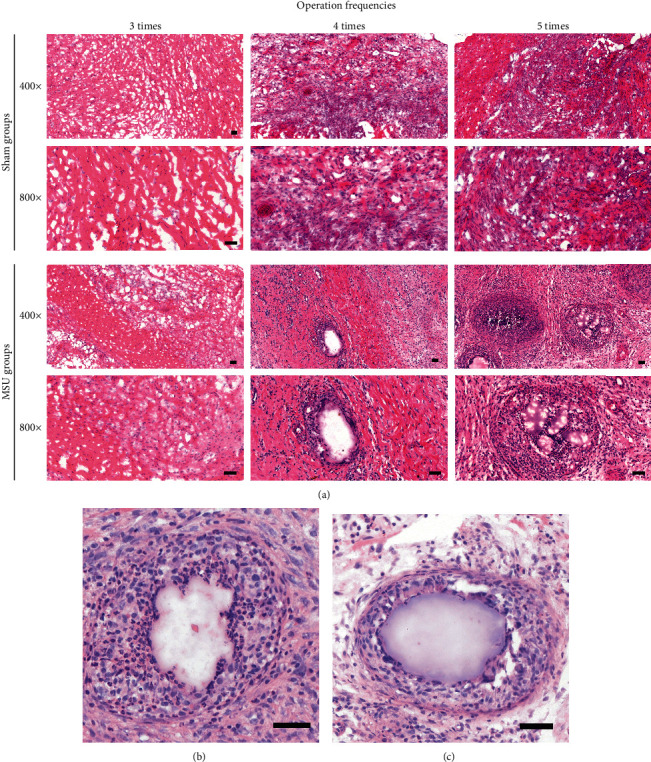
Inflammatory cell infiltration and deposition of MSU in synovial tissue. Representative images of pathological section showed a progressive MSU-mediated inflammation. (a) Top, in the sham groups, the synovial inflammation and disordered collagenous fibers were observed. Bottom, MSU deposited in tissues mediating the immune response and recruiting a large number of inflammatory cells. MSU deposition was surrounded by the inflammatory cells and collagen fibers. (b, c) Large-scale images of MSU deposition surrounded by inflammatory cells in MSU groups received 4-5x interventions. Deposition of MSU was mainly surrounded by lobulated neutrophils. Scale bars, 50 *μ*m.

The histopathological differences between the MSU group and the sham group were demonstrated by H&E staining. In the saline groups, simple synovitis presented as inflammatory cell infiltrations. In the MSU intervention group, the sections showed serious synovial hyperplasia, cell proliferation, and collagen fiber disorder increased with the frequencies of interventions (Figure 4(a)). Different from the previous acute gout arthritis model, recruited neutrophil packed with MSU crystal deposition was observed in the MSU group. The H&E stain clearly demonstrates the eosinophilic acellular structure (MSU crystals) and multiple surrounding layers of lobulated neutrophils (Figure 4(b)).

**Figure 5:**
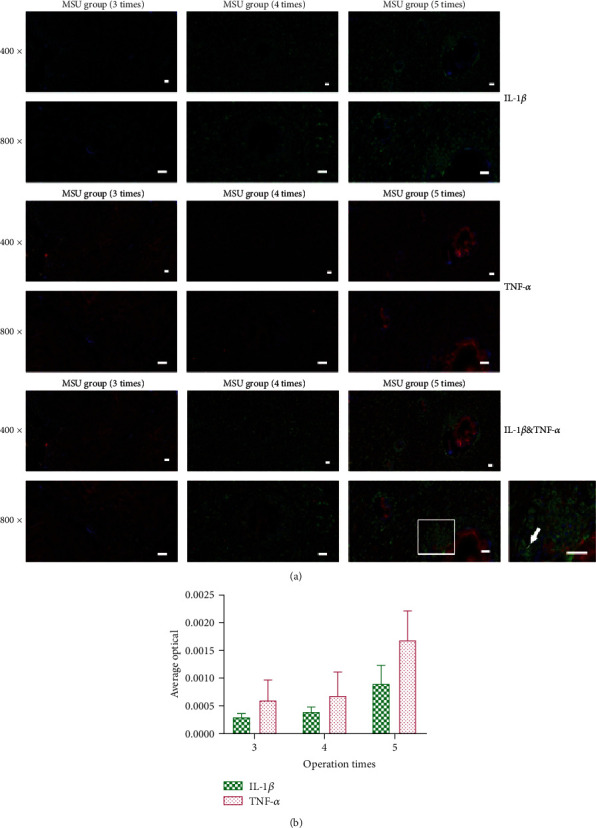
Immunofluorescence revealed upregulation of IL-1*β* expression in the MSU embedding rat model. (a) Representative images of immunofluorescence showed regulation of cytokine expression in MSU embedding groups. IL-1*β* (green) expression was significantly upregulated in tissues. Similarly, TNF-*α* (red) is also highly expressed around MSU deposition. The arrow indicates a significant expression of IL-1*β* in cells surrounding MSU. (b) Further quantitative average optical (AO) analysis showed that the expression levels of two cytokines were upregulated with the increase of intervention frequencies (*n* = 4 for each group; one-way ANOVA and Tukey's multiple comparisons tests were applied for each cytokine). Data shown as the mean ± SD. Scale bars, 50 *μ*m.

Immunofluorescence revealed the expression level of inflammatory factors in synovial tissue after MSU implantation. IL-1*β* and TNF-*α* are highly coexpressed in aggregated inflammatory cells (Figure 5(a)). Further quantitative average optical (AO) analysis showed that the expression levels of two cytokines were upregulated with the increase of intervention frequencies, which also demonstrated the typical MSU-mediated synovial inflammation *in vivo* (Figure 5(b)).

## 4. Discussion

This study designed a reliable and economical animal model for persistent gout arthritis manifested by significantly prolonged gross and behavioral abnormalities, as well as intra-articular MSU deposition and the tophi formation.

Animal models of gout are necessary basis for researchers to understand the disease progress and to determine potential treatments and prevention measures. A literature search based on the PubMed database was conducted among studies of gouty arthritis over the past decade. More than 120 articles are applying the injection model to study the mechanism of inflammatory cytokines regulation or acute inflammatory responses. Nevertheless, uric acid oxidase is lost in human but presenting in most mammals (rats, rabbits, etc.), which causes that serum uric acid levels of other mammals are only one-tenth of human [23, 24]. Hence, a limited dose of MSU injection often causes acute joint inflammation, while the threshold of tophi formation and crystal deposition is rarely reached. Without appropriate animal model, few studies are available regarding the formation, growth, attachment, and deposition of MSU as well as interaction between crystals and joint tissue *in vivo* [25].

The present study puts accurate repeatable implantation of larger doses of MSU into the articular cavity and maintains a continuous high concentration of MSU in the articular cavity to resist the effect of uric acid oxidase. Pain and joint swelling are typical symptoms of gout arthritis. In the injection model, local symptoms are proved to present as self-limiting and relive within 72 hours [16]. Consistently, the reduction of the PWT in MSU injection models was reported to maintain within 48 to 72 hours in previous researches [16]. In contrast, this study significantly prolonged the persistence of local symptoms, with significant synovial swelling and mechanical hyperalgesia more than five days after interventions. This outcome is caused by MSU embedding rather than surgical incision due to the presence of a control group. In terms of pathology, MSU crystal deposition was observed surrounded by IL-1*β* and TNF-*α*-positive inflammatory cell. This is the first report of exogenous MSU deposition in synovium in animal models into providing potential tools for clinical identification and debridement. The model also provides an ideal approach for further studying the pathophysiological changes of MSU deposition *in vivo* and accurate removal of MSU deposition in the tissue.

Compared with the injection modeling method, more persistent joint inflammation and crystal deposition are observed in this study for three main reasons, including higher dose, more accurate implantation, and combination with artificial defect. (1) The implantation dose was significantly more sufficient than the traditional injection method. Injection dose was distributed between 0.5 mg and 5.0 mg in previous research [12, 15, 16, 26], while the total embedding dose of MSU in this experiment reached 60 to 100 mg. High-dose MSU intervention, though larger than other injection models, was shown to be safe, and no lesions or symptoms outside the joint were found. The results proved that MSU crystals were deposited in the synovium by inflammatory cells before being decomposed by uricase. (2) The space of rats' knee articular cavity is limited with multiple contents including the ligaments, cartilage, and meniscus. In addition, saturated MSU crystals tend to precipitate at room temperature and then block injection needles. Swell and effusion of tissues induced by MSU could further aggravate the stenosis of cavity and increase the failure rate of repeated injection. Minimally invasive approach solved this problem by exposing the joint cavity and locating position of MSU crystals accurately. (3) Previous studies suggested that physic impact would result in traumatic arthritis in rabbits [27, 28]. A radiographic study also found a strong relationship between bone erosion and tophi formation [29]. Therefore, an impaired cartilage accelerates the establishment of a gouty arthritis model and then shortens the trial period [30].

There are some limitations in the present study as follows: (1) the procedures of this minimally invasive operation required practice and repetition in order to get a satisfied result of MSU embedding. Basic surgical training is required to meet the operational requirements. (2) The duration of this model was still much less than the natural course of chronic gouty arthritis in human lasting for a few years. Further studies have to include a more convenient way to embed MSU and increase the sessions of embedment to extend the affect time. It is not verified how long the model will last without further MSU embedding, which will be one of the research directions in the future. (3) In this study, synovial crystal deposition was observed, but the tendon, ligament, cartilage, or bone was not involved. Due to the process of section decalcification, some technical difficulties of preserving crystals from strong acid need to be overcome. Sections of osteochondral specimens should also be analyzed in further researches.

## 5. Conclusion

A minimally invasive surgical method was developed to establish the novel rat model of chronic gouty arthritis. This model was proved to be a simple repeatable method and to mimic the pathological characteristics of human persistent gouty arthritis.

## Data Availability

The authors declare that all experimental data of this study are available by contacting the corresponding author.
